# Benefit of diverse surgical approach on short-term outcomes of MEN1-related hyperparathyroidism

**DOI:** 10.1038/s41598-020-67424-5

**Published:** 2020-06-30

**Authors:** Hye Ryeon Choi, Sun Hyung Choi, Soon Min Choi, Jin Kyong Kim, Cho Rok Lee, Sang-Wook Kang, Jandee Lee, Jong Ju Jeong, Kee-Hyun Nam, Woong Youn Chung, Seunghyun Lee, Namki Hong, Yumie Rhee

**Affiliations:** 1 Department of Surgery, Yonsei University College of Medicine, 50 Yonsei-ro, Seodaemun-gu, 03722 Seoul, Republic of Korea; 2 Department of Internal Medicine, Yonsei University College of Medicine, 50 Yonsei-ro, Seodaemun-gu, 03722 Seoul, Republic of Korea

**Keywords:** Endocrinology, Endocrine system and metabolic diseases

## Abstract

Surgical excision is the preferred treatment for multiple endocrine neoplasia type 1 (MEN1)-related primary hyperparathyroidism (PHPT), although controversy regarding the surgical strategy exists. We retrospectively investigated the short-term outcomes of PHPT by various surgical extents. Thirty-three patients who underwent parathyroidectomy due to MEN1-related PHPT at Yonsei Severance Hospital between 2005 and 2018 were included (age [mean ± SD], 43.4 ± 14.1 [range, 23–81] years). Total parathyroidectomy with auto-transplantation to the forearm (TPX) was the most common surgical method (17/33), followed by less-than-subtotal parathyroidectomy (LPX; 12/33) and subtotal parathyroidectomy (SPX; 4/33). There was no postoperative persistent hyperparathyroidism. Recurrence was high in the LPX group without significance (1 in TPX, 2 in SPX, and 3 in LPX, *p* = 0.076). Permanent and transient hypoparathyroidism were more common in TPX (n = 6/17, 35.3%, *p* = 0.031; n = 4/17, 23.5%, *p* = 0.154, respectively). Parathyroid venous sampling (PVS) was introduced in 2013 for preoperative localisation of hyperparathyroidism at our hospital; nine among 19 patients operated on after 2013 underwent pre-parathyroidectomy PVS, with various surgical extents, and no permanent hypoparathyroidism (*p* = 0.033) or post-LPX recurrence was observed. Although TPX with auto-transplantation is the standard surgery for MEN1-related PHPT, surgical extent individualisation is necessary, given the postoperative hypoparathyroidism rate of TPX and feasibility of PVS.

## Introduction

Multiple endocrine neoplasia type 1 (MEN1) is a rare, hereditary, multiple endocrine tumour syndrome, mainly manifesting as primary hyperparathyroidism (PHPT) in 90% of patients^1^. Surgical excision of the affected parathyroid glands is the treatment of choice for MEN1-related PHPT^2^. The ultimate purpose of parathyroidectomy is to normalise serum calcium levels. However, the optimal extent of parathyroidectomy remains controversial, with most surgeons advocating for total parathyroidectomy (TPX) with heterotopic auto-transplantation or subtotal parathyroidectomy (SPX; 3–3.5 glands).

Heterogeneity of the parathyroid glands in MEN1 patients has been widely investigated^3,4^, and the findings emphasise the need for individualised minimal surgery. Less-than-subtotal parathyroidectomy (LPX) has increasingly gained support for its lesser associated incidence of postoperative permanent hypoparathyroidism, which enables improved quality of life in selected patients^5,6^. A precise preoperative localisation technique is crucial for successful LPX. Several studies have reported the efficacy of preoperative ultrasound(US), ^99m^Tc-MIBI SPECT/CT (^99m^Tc-methoxyisobutylisonitrile single photon emission computed tomography/computed tomography), or 4-dimensional computed tomography (4DCT) for a targeted approach^1,5^. Unlike the above mentioned preoperative localisation techniques, parathyroid venous sampling (PVS) is an invasive technique that has mainly been used in patients with ambiguous results on preoperative localisation studies or persistent hyperparathyroidism after initial surgery^7,8^. However, a role for PVS in initial surgery is emerging^9,10^. In MEN1-related PHPT, the feasibility of PVS for preoperative localisation needs to be clarified.

In this study, we compared the short-term surgical outcomes of MEN1-related PHPT according to various surgical extents at a single tertiary-care hospital. We described our early results with the use of preoperative PVS.

## Material and methods

We retrospectively included 33 patients who underwent parathyroidectomy for MEN1-related PHPT at Yonsei Severance Hospital (Seoul, Korea) between 2005 and 2018. All patients had a postoperative follow-up of at least 12 months. Thirty-two patients had been diagnosed by MEN1 gene mutation whereas one patient was diagnosed by a first-degree family history of MEN1 with more than two of the major clinical manifestations of MEN1^2^ (e.g., primary hyperparathyroidism, pituitary adenoma, and enteropancreatic tumour). Clinicopathologic factors such as age, sex, MEN1 gene mutation status, family history of MEN1, laboratory results, findings of preoperative imaging study, and pathology of excised parathyroid glands were reviewed from patient medical records and pathology reports.

Patients were divided into three groups by the surgical extents, which were preoperatively and intraoperatively determined by the surgeon. Thymectomy was performed only when inferior parathyroid glands were not clearly identified during neck exploration. Frozen section of excised specimen during operation for confirm the diagnosis was made in such casese. Patients with bilateral lesions on preoperative studies underwent bilateral neck exploration; while patients with four or more enlarged glands in the operative field underwent TPX and heterotopic auto-transplantation. Patients with a history of previous parathyroidectomy underwent TPX for complete resection of all remnant parathyroid glands followed by auto-transplantation. For auto-transplantation, the surgeon selected a gland with minimal hyperplastic changes from among the resected specimens and preserved it in cold saline in a sterile manner during the parathyroidectomy. After parathyroidectomy, the preserved parathyroid gland was minced into small pieces and injected into the brachioradialis muscle of the non-dominant forearm. Patients who preoperatively and intraoperatively had three enlarged glands underwent SPX. The whole or half of a single normal gland was marked with a clip and left in situ. LPX was conducted when there was definite evidence of two or less affected glands.

**Figure 1:**
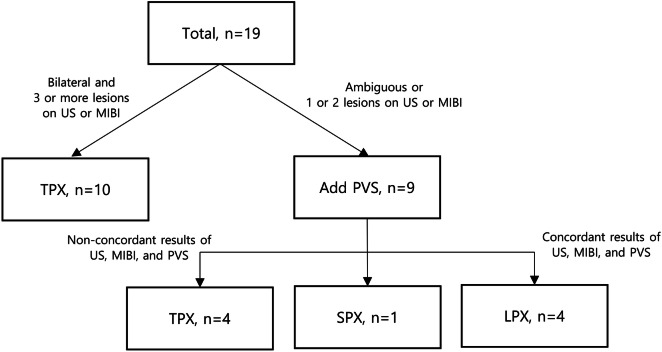
Schematic representation of decision making of the surgical extent after the introduction of parathyroid venous sampling (PVS). TPX, total parathyroidectomy with auto-transplantation to the forearm; SPX, subtotal parathyroidectomy; LPX, less-than-subtotal parathyroidectomy; MIBI, ^99m^Tc-methoxyisobutylisonitrile single photon emission computed tomography; US, Ultrasonography.

Since 2013, PVS has been added to the preoperative localisation study protocol. Figure 1 shows the flow of examination and decision making for the surgical extent.

Ten patients who had confirmed bilateral presence of three or more lesions via US and MIBI scans did not undergo PVS and directly underwent TPX. Nine patients with ambiguous results or one or two lesions on US and MIBI scans underwent PVS. LPX was undertaken when results of all three localisation studies were concordant. If the results of the studies were not concordant or showed bilaterality with three or more lesions, then TPX was performed.

Intraoperative parathyroid hormone (PTH) monitoring was undertaken in all patients except in two of the early cases. Blood samples were obtained from the radial arterial line 5–15 min after the delivery of each specimen. A more than 50% decrease of PTH from the baseline value (preoperative PTH measured on the day before surgery) was considered as the criteria for termination of surgery^11^. Definitions of recurrence, persistent hyperparathyroidism, and transient and permanent hypoparathyroidism were adopted from Horiuchi et al.^12^ Both normocalcemic and hypercalcemic high PTH levels occurring after a minimum of 6 months of the normal postoperative period were defined as recurrence, whereas persistent disease was defined as non-normalisation of PTH and serum calcium postoperatively. The cut-off value between transient and permanent hypoparathyroidism after surgery was 6 months.

A chi-squared or Fisher’s exact test was used to compare categorical data. For continuous data, an ANOVA or Kruskal–Wallis test was used. All data were analysed with IBM SPSS Statistics ver. 22.0 (IBM, Armonk, NY, USA). A *p*-value < 0.05 was considered statistically significant. The study protocol was approved by the institutional review board (IRB) of the Yonsei University College of Medicine (IRB No. 4–2020-0,014). The research was performed in accordance with the Declaration of Helsinki. Informed consent was obtained from all patients.

## Results

Patient characteristics are presented in Table 1. There were no intergroup differences in age and follow-up period among the three groups. Preoperative intact PTH level was highest in the TPX group (*p* = 0.017), followed by those in the SPX and LPX groups. There were 26 germline mutations in a total of 32 patients. Most mutations were found in exon 2 (11/32, 34.38%).

**Table 1:** Patients characteristics.

	Total	TPX	SPX	LPX	*p* value
No. of patients	33	17	4	12	
Age (y)	43 (43.4 ± 14.1)	47 (48.0 ± 16.49)	41 (42.0 ± 10.8)	36 (37.4 ± 8.9)	0.135
Follow-up period (m)	58 (65.2 ± 42.5)	42 (47.4 ± 19.8)	127 (109.0 ± 70.1)	83 (76.0 ± 45.0)	0.130
Preoperative Ca (mg/dL)	10.7 (10.7 ± 0.7)	10.9 (10.7 ± 0.8)	10.7 (11.1 ± 0.8)	10.6 (10.6 ± 0.6)	0.522
Preoperative iPTH (pg/mL)	147.1 (154.6 ± 79.8)	154.5 (190.9 ± 90.6)	140.1 (138.1 ± 52.2)	104.1 (108.8 ± 37.7)	0.017*
Genetically confirmed	31 (93.9%)	17 (100%)	3 (75%)	11 (91.7%)	0.227
Other MEN1 Sx	28 (84.8%)	16 (94.1%)	4 (100%)	8 (66.7%)	0.131
No. preoperative -detected parathyroid	2.0 (1.9)	2.0 (2.1 ± 0.7)	3.0 (2.8 ± 0.5)	1.0 (1.4 ± 0.5)	0.001*
No. intraoperative-confirmed abnormal parathyroid	3.0 (3.0)	4.0 (4.1 ± 0.9)	3.3 (3.3 ± 0.3)	1.5 (1.5 ± 0.5)	0.000*
No. pathologically confirmed adenoma/hyperplasia	4.0 (3.0)	4.0	3.0 (2.8 ± 1.3)	1.0 (1.4 ± 0.5)	0.000*
Auto-transplantation	17 (51.5%)	17 (100%)	0 (0)	0 (0)	0.000*
Thymectomy	11 (33.3%)	10 (58.8%)	0 (0)	1 (9.1%)	0.004*

Data are presented as median or mean ± SD, *p < 0.05.

*TPX* total parathyroidectomy, *SPX* subtotal parathyroidectomy, *LPX* less-than-subtotal parathyroidectomy.

Other MEN1 Sx includes pancreatic neuroendocrine tumour and pituitary adenoma.

Surgical outcomes are presented in Table 2. There was no persistent hyperparathyroidism after surgery. All instances of transient and permanent hypoparathyroidism occurred in the TPX group. Among six patients who developed postoperative permanent hypoparathyroidism, three recovered within 35 months after surgery (recovery period range: 19–35 months). The number of recurrences was highest in the LPX group but was not statistically significant (*p* = 0.076).

**Table 2:** Surgical outcomes according to the surgical extent.

	Total (n = 33)	TPX (n = 17)	SPX (n = 4)	LPX (n = 12)	*p* value
Persistent hyperparathyroidism	0	0	0	0	NA
Transient hypoparathyroidism	4 (12.1%)	4 (23.5%)	0	0	0.154
Permanent hypoparathyroidism	6 (18.2%)	6 (35.3%)	0	0	0.031*
Recurrence	6 (35.3%)	1 (5.9%)	2 (50%)	3 (25%)	0.076

*TPX* total parathyroidectomy with auto-transplantation to the forearm, *SPX* subtotal parathyroidectomy, *LPX* less-than-subtotal parathyroidectomy.

Considering these outcomes together, TPX had a significantly high complication rate (*p* = 0.001). All recurrences in the SPX and LPX groups occurred in glands that were not resected. In one case of TPX, a rising trend of PTH without definite suspicious imaging findings was noted. Recurrence-free survival of SPX and LPX was 55 and 44 months, respectively. All intraoperative quick PTH levels measured at 15 min after the delivery of the last specimen were in the normal range, regardless of the surgical extent of resection. Although the mean PTH level was highest in the LPX group, this was not statistically significant (*p* = 0.357).

In 2013, PVS was introduced at our hospital. PVS was performed to patients with one or two enlarged parathyroid glands identified on preoperative US and ^99m^Tc-MIBI SPECT/CT. Consistent results of all three of preoperative localisation studies were considered as an appropriate incdication for LPX. Table 3 shows the detailed results of preoperative localisation studies, surgical extent and its associations with pathology in PVS group.

**Table 3:** Detailed description of preoperative localisation studies and pathological results.

	Lesions found at US & MIBI	Lesions found at PVS	Surgical extent	Pathologically positive lesion
Case 1	Rt. Superior	Rt. superior	TPX	Rt. superior
		Rt. Inferior		
	Lt. Superior			Lt. superior
		Lt. inferior		
Case 2		Rt. superior	TPX	Rt. superior
	Rt. inferior	Rt. inferior		Rt. inferior
	Lt. superior			Lt. superior
				Lt. inferior
Case 3		Rt. superior	TPX	Rt. superior
				Rt. inferior
	Rt. Inferior	Rt. inferior		Lt. inferior
		Lt. superior		
		Lt. inferior		
Case 4		Rt. superior	TPX	Rt. superior
		Rt. inferior		Rt. inferior
	Lt. superior	Lt. superior		Lt. superior
	Lt. inferior	Lt. inferior		
Case 5	Rt. superior	Rt. superior	SPX	Rt. superior
		Rt. inferior		Rt. inferior
	Lt. superior	Lt. superior		Lt. superior
Case 6	Ambiguous	Rt. superior	LPX	Rt. superior
Case 7	Rt. inferior	Rt. inferior	LPX	Rt. inferior
Case 8	Rt. superior	Rt. superior	LPX	Rt. superior
	Rt. inferior	Rt. inferior		Rt. inferior
Case 9	Rt. inferior	Rt. inferior	LPX	Rt. inferior
	Lt. superior	Lt. superior		Lt. superior

*TPX* total parathyroidectomy with auto-transplantation to the forearm, *SPX* subtotal parathyroidectomy, *LPX* less-than-subtotal parathyroidectomy, *MIBI* ^99m^Tc-methoxyisobutylisonitrile single photon emission computed tomography, *US* ultrasonography, *PVS* parathyroid venous sampling, *Rt.* right, *Lt.* left.

Table 4 presents the results of preoperative PVS; the PVS group showed no persistent hyperparathyroidism or permanent hypoparathyroidism postoperatively. There was no recurrence even after LPX.

**Table 4:** Surgical outcomes according to preoperative parathyroid venous sampling.

	PVS (n = 9)	Non-PVS (n = 10)	*p* value
Persistent hyperparathyroidism	0	0	NA
Transient hypoparathyroidism	2 (22.2%)	1	0.582
Permanent hypoparathyroidism	0	5 (50%)	0.033*
Recurrence	0	1 (10%)	1.000
TPX	4 (44.4%)	10 (100%)	0.011*

*PVS* parathyroid venous sampling, *TPX* total parathyroidectomy with auto-transplantation to the forearm.

## Discussion

MEN1 is caused by a germline-inactivating mutation of the tumour suppressor gene, *MEN1*, located on chromosome 11q13, which encodes the menin protein, an important transcriptional regulatory factor^13,14^. There are more than 1,000 known germline mutations that can cause MEN1^14^. As every gland has the same potential risk of hyperplasia due to those genetic alterations, TPX with heterotopic auto-transplantation is considered the eventual surgical method for MEN1-related PHPT.

TPX may be the only curative surgery for MEN1-related PHPT; however, permanent hypoparathyroidism is a major complication that is more troubling than disease recurrence. Nonetheless, there is no effective way to intraoperatively evaluate the viability of the auto-transplantated parathyroid gland. SPX was proposed as a feasible alternative method to replace TPX^15^. However, the rate of permanent hypoparathyroidism after SPX has not improved significantly compared to that after TPX^1^. The range of permanent hypoparathyroidism in the SPX group was reported as 0–40%^12^. Lorente-Poch at el. reported that auto-transplantation of the accidentally resected fourth parathyroid gland could not prevent the occurrence of permanent hypoparathyroidism in total thyroidectomy^16^. The risk of permanent hypoparathyroidism after total thyroidectomy is well known. Bergenfelz et al. and Almquist et al. reported that permanent hypoparathyroidism was not only associated with an increased risk of renal insufficiency, malignancies, and cardiovascular events but also with increased mortality^17,18^. Although these studies were conducted on patients with normal parathyroid glands, a similar pathophysiology may be applied to parathyroidectomy after MEN1-related hyperparathyroidism.

The concept of limited surgery in MEN1-related PHPT was presented to overcome the permanent hypoparathyroidism after TPX. This first originated from the premise that all glands are not simultaneously enlarged, and it may not be necessary to eliminate all the glands at once. The size heterogeneity of parathyroid glands in MEN1 is well known^3^. This hypertrophy of glands is associated with hyperfunction. In our study, preoperative PTH was highest in the TPX group, which had the largest number of enlarged parathyroid glands identified, followed by the SPX and LPX groups. This phenotypic asymmetricity can be explained by the independent occurrence of a “second hit” somatic mutation of the MEN1 gene in the four parathyroid glands^1^. Marini et al. reported incomplete penetrance of menin mutations. They analysed 410 MEN1 patients including 370 familial cases. Despite the presence of the same genetic mutation within the same pedigree, patients showed varying degree of severity of clinical symptoms^19^. Pardi et al. also found an absence of genotype–phenotype correlation in MEN1^20^. These findings suggest that patients can be categorised by the number of preoperatively enlarged parathyroid glands, which is correlated with disease severity.

In selected patients with a limited number of enlarged glands, less aggressive surgery may be sufficient. Several reports support this theory, and conclude that the excision of only enlarged glands could achieve long-term recurrence-free survival^6,21^. As MEN1-related PHPT is known to develop at a younger age than sporadic PHPT^2^, this is an especially important result for younger patients. A focused approach is known to not only reduce the operation time, incision size, hospital stay, and postoperative pain, but also to lower the incidence of postoperative permanent hypocalcemia^22^. The results of our data are consistent with previous studies. When LPX was conducted in appropriately selected patients, it showed the least incidence of transient or permanent postoperative hypoparathyroidism, with acceptable recurrence rates compared with that of TPX or SPX. The intraoperative PTH levels normalised in all patients after the excision of enlarged glands and there was no persistent hyperparathyroidism.

In addition to the low permanent hypoparathyroidism rate, LPX has another strong advantage. Considering the higher risk of reoperation in patients with MEN1 than in patients with sporadic primary hyperparathyroidism, limited exploration has the benefit of facilitating future surgery via preservation of an unresected area. Blind bilateral neck exploration can cause wide extensive postoperative adhesion, which poses difficulties to reoperation. In our data, all the recurrences of the SPX and LPX groups occurred in unresected glands. A previously untouched operation field made it easy to excise the remnant enlarged glands and there was no reoperation-related recurrent laryngeal nerve injury or prolongation of operation time, compared to the initial surgery (data not shown). In clinical practice, a ‘staged operation’ followed by initial LPX may be a more appropriate concept for MEN1-PHPT in select patients given the balance between potential risks of recurrence and consequences of permanent hypoparathyroidism^6^.

For successive LPX, the selection of indicated patients is important. Elaboration of preoperative imaging techniques has been largely implicated in the increased success rate. Thus far, ^99m^Tc-MIBI SPECT/CT scans have been the modality of choice. However, ^99m^Tc-MIBI SPECT/CT has several limitations. False-negative or positive results can occur due to small parathyroid adenomas, multiglandular disease, or the presence of thyroid nodules^23^. High-resolution US is useful to understand the anatomic relation between parathyroid glands and the adjacent thyroid gland. Although US alone is not enough for preoperative evaluation due to its low sensitivity and specificity^24^, a combination of ^99m^Tc-MIBI SPECT/CT and US has been a popular method for preoperative localisation of PHPT.

However, surgeons hesitate to plan LPX for MEN1 patients even when both US and MIBI have identified one or two enlarged glands. With PVS, surgeons can decide on LPX with greater confidence. In our study, concordant results of ^99m^Tc-MIBI SPECT/CT, US, and PVS were highly predictive for successful LPX. Four patients who had undergone LPX would have undergone TPX if they were operated on prior to 2013. By adding PVS for preoperative localisation and intraoperative quick PTH monitoring for intraoperative evaluation for determine the termination of surgery, the incidence of persistent hyperparathyroidism after LPX can be effectively lowered. PVS was also useful in identifying parathyroid lesions which were missed at previous conventional preoperative imaging studies. In four of five non-concordant cases, PVS detected more lesions than US and ^99m^Tc-MIBI SPECT/CT. Six of nine additional lesions newly found at PVS was pathologically positive for parathyroid adenoma or parathyroid hyperplasia. Lee et al. also reported the discriminative power of PVS for localise parathyroid lesions in patients with negative findings in conventional US and ^99m^Tc-MIBI SPECT/CT^10^.

The most important concern of PVS is radiation exposure. According to our data, the radiation exposure of PVS ranges from 1.23 to 5.8 mSv, which was half of that from 4DCT (10.4 mSv) and lower than MIBI (6.7–7.8 mSv)^10^.

This study has several limitations. It was conducted in a retrospective manner with a limited number of patients. MEN1 is a rare disease, so only 33 patients were treated at our hospital over the past 13 years, despite our hospital being a large tertiary referral center for endocrine surgery. This is not very different from other published data(24–28 patients, over 20–28 years)^5,6,16^. The median follow-up period was relatively short (58 months). Time to recurrence after parathyroidectomy varies among reports, from 1 to 26years^5,6,25^. Although our patients might eventually recur with time, this study showed the benefit of LPX in managing current hyperparathyroidism while avoiding or defer permanent hypoparathyroidism. Our study supports the trend of paradigm shift of reoperation in MEN1-related hyperparathyroidism patients from ‘surgical failure’ to ‘strategic staged operation’. With longer follow-up periods, recurrence free survival of LPX group in our data(44 months) is expected to be changed. Further consensus is needed on how long the period without recurrence after surgery should be achieved to determine LPX as an feasible procedure for MEN1-related PHPT. The surgical extent was decided differently by the surgeon’s preference. The time gap between the introduction of PVS could be problematic. Nonetheless, this is the first study to have evaluated the efficacy of PVS in MEN1-related PHPT. Thus, adding PVS in selected patients with MEN1-related PHPT may be helpful to decide on safe and effective limited parathyroidectomy. These findings need to be validated in future larger sample studies.

## Conclusion

TPX with auto-transplantation has been considered the standard surgical treatment for MEN1-related PHPT. However, considering the significant postoperative hypoparathyroidism rate associated with TPX and the feasibility of PVS, individualised decisions regarding the surgical extent of resection in MEN1-related PHPT is important.
